# Fecal Microbial Community Characteristics of Oula and Hu Sheep and Their Correlation with Semen Quality

**DOI:** 10.3390/ani16060953

**Published:** 2026-03-18

**Authors:** Lu Shao, Peidi Zhao, Jiaxun Dong, Xiuxiu Weng, Wanhong Li

**Affiliations:** State Key Laboratory of Herbage Improvement and Grassland Agro-Ecosystems, College of Pastoral Agriculture Science and Technology, Lanzhou University, Lanzhou 730020, China; shaol2024@lzu.edu.cn (L.S.); zhaopd2023@lzu.edu.cn (P.Z.); dongjx2024@lzu.edu.cn (J.D.); wengxx@lzu.edu.cn (X.W.)

**Keywords:** fecal microbiota, Oula sheep, Hu sheep, Qinghai–Tibet Plateau, semen quality

## Abstract

Semen quality and fecal microbial composition were compared between native Oula rams reared on the Qinghai–Tibet Plateau and Hu sheep rams introduced from lowland regions. Semen quality was analyzed in eight adult Oula rams and eight Hu rams, and fecal microbial composition was assessed via 16S rRNA sequencing. Results indicated that sperm acrosome integrity was significantly higher in Hu sheep than in Oula sheep (*p* < 0.001); other semen parameters showed no significant differences. Significant differences were also observed in fecal microbial communities between the two breeds. Compared with Hu sheep, Oula sheep exhibited higher microbial abundance and diversity at the phylum level, particularly Campylobacterota, Euryarchaeota, Planctomycetota, Verrucomicrobiota, Myxococcota, and Deferibacterota (*p* < 0.05). At the genus level, Oula sheep had significantly higher abundances of *Treponema*, *Campylobacter*, *Methanobrevibacter*, UCG-009, Family_XIII_AD3011_group, [*Eubacterium*]*nodatum* group, *Candidatus Soleaferrea*, *Akkermansia*, and *unidentified_Ruminococcaceae* (*p* < 0.05). Correlation analysis indicated associations between sheep semen quality and the top 30 abundant fecal microbial genera. Six genera showed significant positive correlations with acrosome integrity rate, and eight genera exhibited significant negative correlations (*p* < 0.05). Two genera were correlated positively with plasma membrane integrity rate (*p* < 0.05). *Prevotellaceae_*UCG-004 was positively correlated with sperm motility and Progressive Motility spermatozoa proportion (*p* < 0.05); *Ruminococcus* showed a significant positive correlation with sperm linear motility and a significant negative correlation with acrosome integrity rate (*p* < 0.05). These findings indicate that the microbial groups enriched in Oula sheep fecal samples and exhibiting negative correlations with acrosome integrity—including *Ruminococcus*, *Treponema*, *Akkermansia*, and Euryarchaeota—are associated with sperm quality through physiological adaptation mechanisms specific to high-altitude environments.

## Simple Summary

Gut microbiota plays a crucial role in helping animals adapt to extreme environments, but it is unclear whether this adaptive advantage comes at the cost of reproductive performance. To investigate this issue, this study compared the fecal microbiota and semen quality of locally adapted high-altitude Euler sheep and introduced low-altitude lake sheep raised in the same environment on the Qinghai–Tibet Plateau. Sperm acrosome integrity and related parameters were assessed using the hypo-osmotic swelling test and Giemsa staining, and fecal microbial communities were systematically analyzed via 16S rRNA amplicon sequencing. Results showed that compared with Hu sheep, Oula sheep had significantly higher abundances of fecal microorganisms associated with enhanced fiber degradation, increased volatile fatty acid production, and reduced methane (CH_4_) generation, such as *Akkermansia*, *Treponema*, and *Ruminococcus*. The enrichment of these microbial groups was negatively correlated with sperm acrosome integrity, and this relationship may be associated with high-altitude adaptive regulatory mechanisms affecting reproductive capacity. This study provides theoretical support for the superior adaptability of Oula sheep in high-altitude pastoral environments.

## 1. Introduction

Differences in natural environments and societal demands across regions have led local populations, through long-term selection, to develop sheep breeds exhibiting distinct physical characteristics and physiological traits. In particular, China is home to numerous excellent sheep breeds, including Hu sheep, Mongolian sheep, Tibetan sheep, Oula sheep and Kazakh sheep. The gastrointestinal microbiota has long maintained a mutualistic symbiotic relationship with its host. Environmental variations significantly influence the diversity and composition of the host’s gut microbiota, which contributes to the host’s environmental adaptability [1]. A recent study by Xu et al. [2] demonstrated that host genetics are among the most critical intrinsic factors governing the composition and structure of the gut microbiota. Li et al. [3] revealed that the gut microbiota participates in regulating the intestinal barrier and material metabolism of ruminants, playing a crucial role in their health and production performance. Gastrointestinal microbiota composition varies among different livestock breeds [4]. Cheng et al. [5] compared rumen microbial compositions among sheep breeds from three distinct ecological environments and found that the Hu sheep originating from China’s Taihu Basin were highly clustered in *Prevotella* of Bacteroidota, the Tan sheep from northwestern desert regions in *Methylorubrum* of Proteobacteria, and Dorper sheep introduced from foreign sheep breeds in *Fibrobacter* of Fibrobacterota. Liu et al. [6] compared yaks with Qaidam cattle and found that high-altitude ruminants harbor specialized rumen bacterial communities enriched in fibrolytic and H_2_-incorporating taxa, leading to more efficient fiber digestion and lower methane emissions.

Oula sheep is a Tibetan sheep breed primarily distributed in Gansu and Qinghai provinces along the eastern edge of the Qinghai–Tibet Plateau. They exhibit remarkable adaptability to the harsh plateau environment characterized by high altitude, cold temperatures, low oxygen levels, intense ultraviolet radiation, and limited forage availability. This breed is widely reared by plateau herders because of its outstanding growth rate, meat quality, and tolerance for coarse feed. The gut microbiota influences animal health and production performance by participating in feed digestion and metabolism, host health maintenance, and environmental adaptation. The gut microbiota is increasingly recognized as a key mediator of such environmental adaptation, enabling hosts to optimize energy harvest from poor-quality forage and maintain physiological homeostasis under hypoxic stress [1,7]. However, as China’s meat sheep industry transitions from traditional grazing to intensive, large-scale housing systems, reproductive efficiency has become a pivotal factor determining industrial profitability. Plateau local breeds such as the Oula sheep generally exhibit low reproductive performance, typically producing only one lamb per year and failing to meet the demands for productivity and economic viability in large-scale farming. This phenomenon creates an urgent need to introduce high-fertility local breeds for crossbreeding improvement. Originating from China’s Taihu basin, Hu sheep exhibit high fertility and broad adaptability and have been extensively introduced to other regions of China for breeding and crossbreeding improvement. The phenotypic divergence between these two breeds—Oula sheep exhibiting enhanced high-altitude adaptability and Hu sheep demonstrating superior reproductive performance—suggests an important consideration: whether a microbiota-mediated trade-off exists between environmental adaptation and reproductive investment. Recent studies have suggested that the gut microbiota can remotely regulate host reproductive function through the “gut–reproductive axis,” with potential involvement of systemic energy metabolism, inflammatory responses, and oxidative stress status [8]. In high-altitude environments, hypoxia-induced alterations in gut microbial composition may influence host oxidative status and energy allocation, thereby exerting potential effects on spermatogenesis—a process highly susceptible to oxidative stress [9]. Sperm acrosome integrity, in particular, is highly vulnerable to reactive oxygen species (ROS) attack under conditions of oxidative stress resulting from energy metabolic imbalance, owing to the abundance of polyunsaturated fatty acids in the acrosomal membrane. However, there is currently no evidence directly linking the fecal microbial composition of adult rams to their semen quality in the context of high-altitude adaptation. It remains unknown whether the chronic hypoxic stress on the Qinghai–Tibet Plateau influences the gut–reproductive axis in sheep, and whether a microbiota-mediated trade-off between environmental adaptation and reproductive investment exists in native breeds like the Oula sheep.

Therefore, this study analyzed the semen quality and fecal microbiota of adult Hu and Oula sheep maintained under identical environmental conditions on the Qinghai–Tibet Plateau. We hypothesized that: (1) the unique adaptation strategies of Oula and Hu sheep could potentially shape the composition of their fecal microbiota; and (2) the enrichment of specific microbial taxa in Oula sheep might be associated with a trade-off between adaptation to extreme environments and reproductive performance. This exploratory study aims to provide foundational data and generate testable hypotheses for future mechanistic research on the role of the gut microbiota in mediating host adaptation and reproductive performance in high-altitude ruminants.

## 2. Materials and Methods

### 2.1. Experimental Animals and Sample Collection

The trial was conducted at Gansu Mingxin Sheep Breeding Co., Ltd. (Lanzhou, China). Sixteen adult breeding rams (n = 8 for Hu sheep, 75.45 kg ± 5.44 and n = 8 for Oula sheep, 79.00 kg ± 2.71) aged approximately 2 years, healthy and free from disease, and with normal growth and development were selected. The experimental rams were housed in separate pens under stall-feeding conditions and received total mixed rations at 07:30 and 18:30 daily with ad libitum access to feed and water. Samples were collected in November, corresponding to the physiological period of Oula sheep’s breeding season, while Hu sheep are still in the active breeding season, with no antibiotic use within the preceding 2 months. Ram semen was collected in an indoor sperm collection chamber using the artificial vaginal method. Before collection, all semen contact devices were strictly sterilized and rinsed with 37 °C saline. Five and three days before formal sperm collection, semen was collected once to excrete aged sperm. Each ram was collected twice during sampling for analysis. The relevant parameters were measured immediately after sperm collection in a 37 °C thermostatic water bath. At the same time, fecal samples (approximately 2–3 g per tube) from 16 rams were collected through the rectum into sterile 5 mL centrifuge tubes. The collected fecal samples were immediately placed in liquid nitrogen for transport to the laboratory and subsequently stored at –80 °C until DNA extraction and sequencing analysis.

### 2.2. Semen Quality Analysis

After collecting semen, the ejaculation volume was immediately measured directly from the graduated collection tube and pH test strips were used to determine the pH value of the semen. Sperm concentration (×10^9^/mL) was measured using a hemocytometer (Shanghai Qiujing Biochemical Co., Ltd., Shanghai, China) under light microscopy at 400× magnification (Olympus CX31, Olympus Corporation, Tokyo, Japan); to immobilize sperm for accurate counting, fresh semen was diluted 100-fold with 3% NaCI. After thorough mixing, 10 μL of the diluted sample was loaded onto a hemocytometer, and sperm were counted in five squares. Sperm motility rate (%) and progressive motility (PR, %) were assessed using a computer-assisted semen analysis (CASA) system (ML-800, Xuzhou Qibo Electronic Technology Co., Ltd., Xuzhou, China), where 10 μL of 10-fold diluted semen was examined at 100× magnification across five random fields. Sperm linear motion (μm/s) and curved motion (μm/s) were measured by the CASA (ML-800, Xuzhou Qibo Electronic Technology Co., Ltd.) system, representing the average velocity along a straight line between the first and last detected positions and along the actual trajectory, respectively. Sperm plasma membrane integrity rate [10] was evaluated using the hypo-osmotic swelling (HOS) test, where 20 μL of diluted semen was incubated with 200 μL of hypo-osmotic solution (100 mOsm/L sodium citrate-fructose) at 37 °C for 30 min, and sperm with coiled tails were counted as intact under light microscopy at 200× magnification. Acrosome integrity rate [11] was assessed by Giemsa staining using a commercial Giemsa staining kit (Cat. No. G1010, Beijing Solarbio Science & Technology Co., Ltd., Beijing, China). The working solution was freshly prepared before use by mixing 1 part Giemsa Stock Solution with 9 parts Giemsa Buffer according to the manufacturer’s instructions. Then, 10 μL of 100-fold diluted semen (diluted with distilled water) was smeared on a glass slide and air-dried. The smear was fixed in formaldehyde solution (Shuangshuang Chemical Co., Ltd., Yantai, China) for 2 h, then rinsed gently with running water. After fixation, the slide was immersed in the freshly prepared Giemsa working solution for 2.5 h at room temperature. Following staining, the slide was rinsed gently with running water to remove excess stain and air-dried. The slide was examined under light microscopy at 400× magnification. Sperm with uniformly stained acrosomes (dark blue–purple) were classified as intact, while those with lightly stained or unstained acrosomes were classified as damaged. A total of 200 sperm were counted per sample.

### 2.3. 16S rRNA Gene Sequencing

#### 2.3.1. DNA Extraction and Sequencing

DNA was extracted from samples using the DP328 Fecal Genomic DNA Kit (centrifugation column format). DNA concentration was measured using a UV-visible spectrophotometer (Thermo Fisher Scientific, Inc., Waltham, MA, USA), and DNA integrity was assessed via 0.8% agarose gel electrophoresis. The V4 region was amplified using specific primers (515F-806R; forward primer F: GTGYCAGCMGCCGCGGTAA; reverse primer R: GGACTACNNGGTATCTAAT). Each PCR reaction contained 15 µL of Phusion High-Fidelity PCR Master Mix (Tiangen Biochemical Technology Co., Ltd., Beijing, China), 0.2 µM primers, and 10 ng of genomic DNA template. Initial denaturation occurred at 98 °C for 1 min, followed by 30 cycles at 98 °C (10 s), 50 °C (30 s), and 72 °C (30 s), concluding with a 5 min extension at 72 °C. Libraries were constructed and then quantified using Qubit and Q-PCR. Upon library qualification, PE250 sequencing was conducted using the NovaSeq 6000 platform (Illumina, Inc., San Diego, CA, USA).

#### 2.3.2. Data Quality Control

After barcodes and primer sequences were trimmed, FLASH (Version 1.2.11, https://ccb.jhu.edu/software/FLASH/, accessed on 30 May 2024) was employed to assemble reads from each sample, yielding raw tag data. The assembled raw tags underwent stringent filtering using fastp software (Version 0.23.1) to obtain high-quality tags (clean tags). These tags were then aligned against species annotation databases (Silva database, https://www.arb-silva.de/, Unite database, https://unite.ut.ee), and chimeric sequences were removed to obtain the final effective tags.

#### 2.3.3. ASV Noise Reduction and Species Annotation

For the resulting effective tags, noise reduction was performed using either the DADA2 module or deblur within the QIIME2 software (Version QIIME2-202202), yielding the final amplicon sequence variants (ASVs) and feature table. Species annotation was performed using QIIME2 software. The 16S and 18S databases employed were Silva138.1, and the ITS database utilized was Unite v9.0. For noncanonical regions, annotation was defaulted to the micro_NT database.

#### 2.3.4. Analysis of Fecal Microbiota Community Differences

Further calculations of α-diversity and β-diversity were performed using QIIME2 software. Microbial community structure diversity was assessed by calculating Bray–Curtis distances. Nonmetric multidimensional scaling (NMDS) and principal component analysis (PCA) were conducted using R software (V4.0.3) and implemented via the ade4 (V4.0.3) and ggplot2 (V4.0.3) packages. Differences between high-dimensional datasets were tested using Analysis of Similarity by Multiple Observations (ANOSIM), Analysis of Variance in a Similarity Matrix (ADONIS), and the Multiple Response Permutation Procedure (MRPP) to determine whether intergroup differences were significantly greater than intragroup differences, thereby establishing the validity of grouping. Analyses and visualizations were performed within R (V4.0.3) using the vegan (V4.0.3) and ggplot2 (V4.0.3) packages. Microbial abundance data at the phylum, family, and genus levels for the top 30 most abundant taxa in fecal samples from both sheep breeds were selected. Cluster heatmaps were generated using R’s pheatmap () function. LEfSe software (V1.1.01) was used to analyze biomarkers between groups, producing LDA value distribution bar charts and evolutionary branch diagrams.

#### 2.3.5. Functional Analysis of Fecal Microbiota Differences

Microbial abundance data were obtained for the top 20 phyla, top 20 families, and top 30 genera in the fecal samples from two sheep breeds. Cluster heatmaps were generated using the pheatmap () function in R. LEfSe (V1.1.01) software was employed to analyze biomarkers between the two groups, producing LDA value distribution bar charts and evolutionary branch diagrams. Microbial community functions were predicted using the PICRUSt2 (V1.1.4) tool, integrated with the MetaCyc and KEGG databases. Correlations between key microbial genera and KO (KEGG Orthology) pathways were assessed via Pearson correlation analysis using the R (V4.0.3) package. All correlation results were visualized using the R’s pheatmap (). 

### 2.4. Analysis of Semen Quality and Fecal Microbial Correlations

The top 30 key bacterial genera from both sheep groups were selected. With the use of the pheatmap () function in R (V4.0.3), correlation heatmaps were generated between these key genera and semen parameters, including ejaculate volume, pH, sperm motility rate, progressive motility, sperm linear motion velocity, sperm curved motion velocity, sperm concentration, acrosome integrity rate, and plasma membrane integrity. Significance levels were denoted by asterisks.

### 2.5. Statistical Analysis

Semen quality parameters were statistically analyzed using Excel 2016 software and SPSS 25.0 software. Differences were considered statistically significant at *p* < 0.05.

## 3. Experimental Results

### 3.1. Body Weight and Semen Quality Analysis

**Figure 1:**
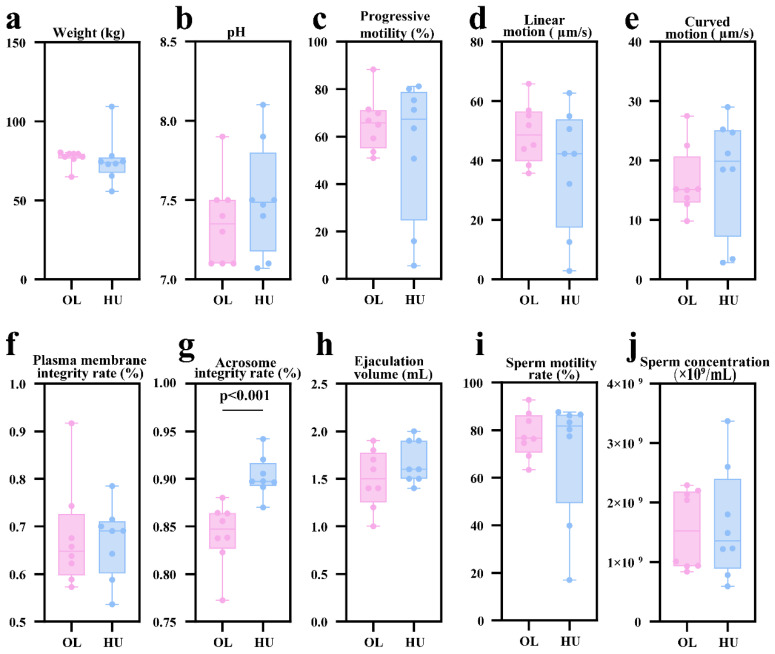
Analysis of body weight and semen quality in Oula sheep (OL) and Hu sheep (HU). (**a**) Body weight; (**b**) Semen pH; (**c**) Progressive motility; (**d**) Sperm linear motion velocity; (**e**) Sperm curved motion velocity; (**f**) Sperm plasma membrane integrity rate; (**g**) Acrosome integrity rate; (**h**) Ejaculate volume; (**i**) Sperm motility rate; (**j**) Sperm concentration.

The results are shown in Figure 1. No significant differences in body weight (Figure 1a), semen pH (Figure 1b), progressive motility (PR, %) (Figure 1c), sperm linear motion (Figure 1d), sperm curved motion (Figure 1e), sperm plasma membrane integrity rate (Figure 1f), ejaculation volume (Figure 1h), sperm motility (Figure 1i) and sperm concentration (Figure 1j) were found between Hu and Oula sheep (*p* > 0.05). However, the sperm acrosome integrity rate (Figure 1g) of Hu sheep sperm was extremely significantly higher than that of Oula sheep (*p* < 0.001).

### 3.2. Sequence Data and Diversity Analysis of Fecal Microbial Samples from Two Sheep Breeds

**Figure 2:**
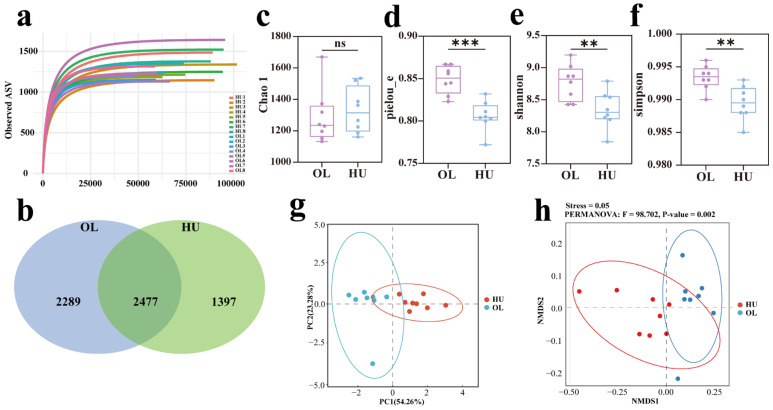
Analysis of rectal fecal diversity and community composition in two sheep breeds. **: *p* < 0.01, ***: *p* < 0.001, ns: The difference is not significant. (**a**) Venn diagram of ASV differences in rectal fecal samples between breeds; (**b**) Dilution curve analysis based on sequence sample size; (**c**) Chao1 index for alpha diversity; (**d**) Simpson index for alpha diversity; (**e**) Shannon index for alpha diversity; (**f**) Pielou_e index for alpha diversity; (**g**) PCA plot based on Bray–Curtis distance; (**h**) NMDS plot based on Bray–Curtis distance, with PERMANOVA results displayed at the top.

Sixteen samples were collected from the feces of two groups of sheep. A total of 1,419,659 raw reads (OL = 6,248,621, HU = 795,038) were obtained from the V4 region (Table 1). Following quality control processing, 594,571 and 740,060 valid sequences were generated for Oula and Hu sheep, respectively. As the number of sequenced reads increased, the sparsity curves for each sample gradually approached saturation, indicating the near-complete detection of the fecal microbial community (Figure 2a). A total of 4766 and 3874 ASVs were annotated exclusively in Oula and Hu sheep, respectively (Figure 2b), with 2477 shared ASVs annotated across both breeds. Alpha diversity results indicated no significant difference in the Chao1 index between the two breeds (Figure 2c). However, the Simpson index (*p* < 0.01), Shannon index (*p* < 0.01), and Pielou_e index (*p* < 0.001) of Oula sheep fecal microbiota were significantly higher than those of Hu sheep (Figure 2d–f). PCA based on Bray–Curtis distance revealed segregation between the two sheep breeds’ fecal microbiota (Figure 2g). Analysis of differences between groups using PERMANOVA (Figure 2h) demonstrated significant differences in the fecal microbial communities of the two breeds (*p* < 0.01). The results were validated using ANOSIM and MRPP to elucidate the significance of fecal microbial community differences between the two breeds (Table 2). The findings confirmed significant structural differences in fecal microbial communities between breeds (*p* < 0.001).

### 3.3. Analysis of Microbial Composition in Fecal Microbiota of Different Sheep Breeds

**Figure 3:**
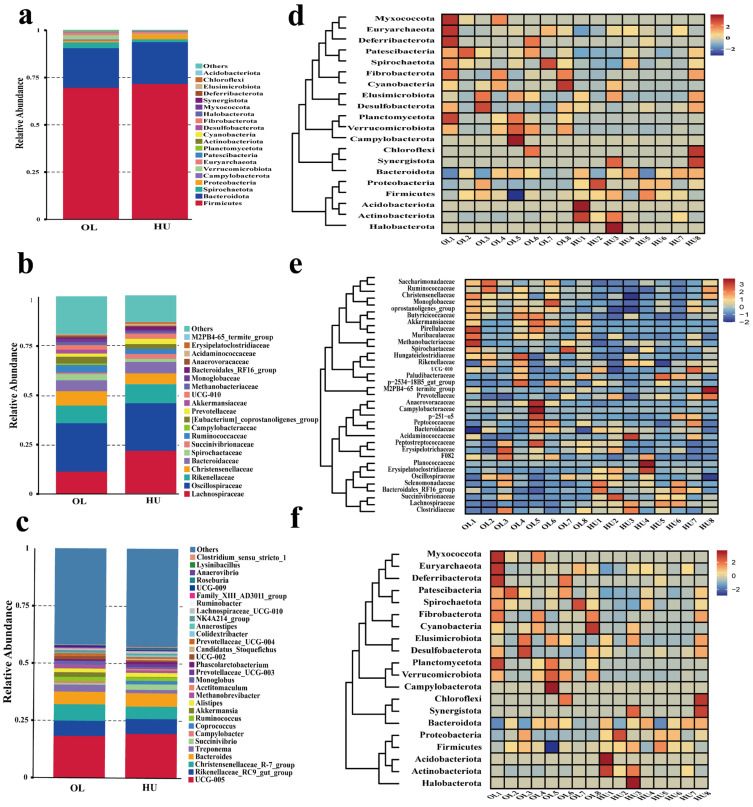
Microbial composition analysis of rectal feces from Oula and Hu sheep. (**a**) Cumulative relative abundance plot of the top 20 microbial species at the phylum level; (**b**) Cumulative relative abundance plot of the top 20 microbial species at the family level; (**c**), Cumulative relative abundance plot of the top 30 microbial species at the genus level; (**d**) Heatmap of the top 20 microbial species clustered at the phylum level; (**e**) Heatmap of the top 20 microbial species clustered at the family level; (**f**) Heatmap of the top 30 microbial species clustered at the genus level, displaying bacterial phylum and genus-level clustering, where color intensity indicates the relative abundance of each bacterial species.

The top 20 species by relative abundance at the phylum and class levels and the top 30 species by relative abundance at the genus level were selected for each group. The remaining species were classified as “Others.” Relative abundance bar charts were plotted for each group’s annotated species across different taxonomic levels. At the phylum level (Figure 3a), Firmicutes and Bacteroidota were the dominant phyla in both breeds, accounting for approximately 70% of the total microbial community, with no significant differences between groups. Among the less abundant phyla, several showed significant differences between breeds (complete list in Figure 3a). Notably, Verrucomicrobiota (containing the mucin-degrading genus *Akkermansia*) and Euryarchaeota (containing methanogens such as *Methanobrevibacter*) were significantly enriched in Oula and Hu sheep, respectively (*p* < 0.05 to *p* < 0.01), both of which are relevant to the hypothesized adaptation–reproduction trade-off. At the family level (Figure 3b), Lachnospiraceae, Oscillospiraceae, Rikenellaceae, and Christensenellaceae constituted the dominant microbial communities for both breeds, accounting for nearly 50% of the total. Among them, Lachnospiraceae, Bacteroidaceae, and Erysipelatoclostridiaceae were significantly lower in Oula sheep than in Hu sheep (*p* < 0.05). Campylobacteraceae, Akkermansiaceae, Methanobacteriaceae, and Butyricicoccaceae were significantly higher in Oula sheep than in Hu sheep (*p* < 0.05). At the genus level (Figure 3c), the dominant genera for both breeds included UCG-005 (an unnamed genus within Firmicutes), *Rikenellaceae_*RC9_gut_group, *Christensenellaceae_*R-7_group, and *Bacteroides*. Compared with Hu sheep, Oula sheep exhibited significantly lower abundances of *Acetitomaculum*, *Candidatus*_*Stoquefichus*, *Anaerostipes*, *Ruminobacter*, and *Lysinibacillus* (*p* < 0.05), while *Coprococcus* was significantly lower in Hu sheep (*p* < 0.01). Conversely, Oula sheep showed significantly higher abundances of several microbial taxa associated with specific functional roles. Fiber degradation-related genera (*Treponema*, *Bacteroides*, *Anaerostipes*, *Ruminobacter*, *unidentified Ruminococcaceae*, *Coprococcus*), mucin-degrading bacterium (*Akkermansia*), *Candidatus_Soleaferrea*, methanogenic archaeon *(Methanobrevibacter*), lipid metabolism-associated genera (*Rikenellaceae_*RC9_gut_group, *Christensenellaceae_*R-7_group), and other bacterial taxa (*Acetitomaculum*, *Campylobacter*, UCG-005, UCG-009, Family_XIII_AD3011_group, [*Eubacterium*]*_nodatum_*group, *Candidatus_Stoquefichus*, *Lysinibacillus*) were significantly enriched in Oula sheep. (*p* < 0.05). Furthermore, the clustering heatmap visually demonstrated distinct microbial abundances and taxonomic groups in the fecal samples of Oula and Hu sheep (Figure 3d–f), revealing significant differences in the fecal microbiota between the two sheep breeds.

### 3.4. PICRUSt2 Microbial Functional Prediction

**Figure 4:**
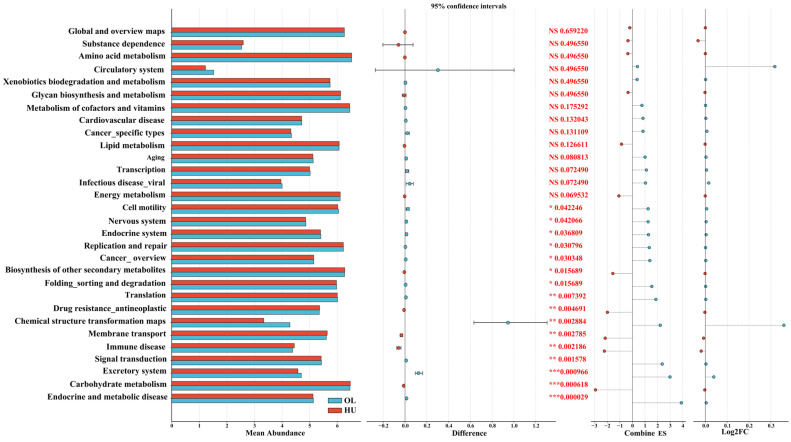
Predicted microbial functions across sheep breeds. The left panel lists multiple KEGG pathways, with the left-hand bar chart displaying the average relative abundance of each pathway in the Hu and Oula sheep groups for visual comparison of overall pathway levels. Differences between groups are presented as scatter plots with 95% confidence intervals to assess statistical significance. The right panel lists the *p*-value for each pathway, annotated with asterisks indicating significance levels (*: *p* < 0.05, **: *p* < 0.01, ***: *p* < 0.001), with nonsignificant pathways marked as “NS.” The combined effect size provides a standardized measure of the magnitude of differences, where high values indicate strong effects. The far-right column, Log2FC (logarithmic fold change), displays the log2-transformed fold change values. Positive values indicate high expression in the Oula sheep group compared with the Hu sheep group, and negative values indicate the opposite.

Microbial functional pathways of different sheep breeds were predicted based on the KEGG database. PICRUSt2 analysis (Figure 4) revealed that the microbial communities in fecal samples from both breeds exhibited enrichment in metabolic pathways, including amino acid metabolism, carbohydrate metabolism, cofactor and vitamin metabolism, polysaccharide biosynthesis and metabolism, energy metabolism, lipid metabolism, and other secondary metabolite biosynthetic pathways. Functional pathways ranked in the top 30 by abundance were analyzed, and the data were subjected to standardization and normalization. PICRUSt2 analysis predicted significant differences in multiple KEGG pathways between the two breeds (Figure 4). Compared to Hu sheep, the Oula sheep fecal microbiome exhibited higher functional abundance in 11 pathways, including those related to “replication and repair,” “translation,” and “folding, sorting and degradation”—processes essential for cellular protein homeostasis and potentially relevant to spermatogenesis, which requires intense protein synthesis and quality control. Additionally, pathways associated with “energy metabolism” and “carbohydrate metabolism” showed differential abundance between breeds. Conversely, the Oula sheep fecal microbiome exhibited significantly lower abundance in five KEGG pathways compared to Hu sheep, including carbohydrate metabolism, biosynthesis of other secondary metabolites, and membrane transport. However, these predictions represent microbial gene potential rather than confirmed host physiological states. 

### 3.5. LEfSe Analysis

**Figure 5:**
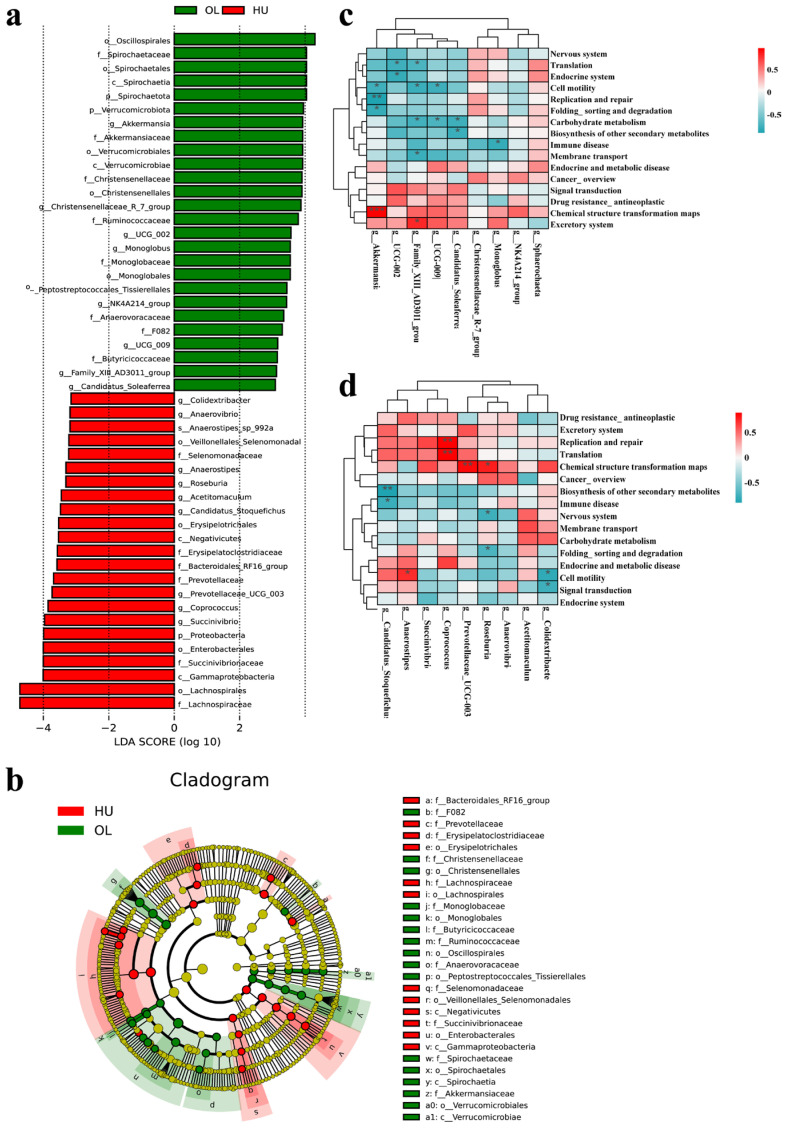
LEfSe illustrates differences in rectal bacterial taxon abundance between Oula and Hu sheep, with significance levels denoted by asterisks (r: correlation coefficient. *: *p* < 0.05, **: *p* < 0.01, ***: *p* < 0.001). (**a**) LDA threshold = 3. LDA score histogram revealing statistically significant biomarkers between groups. Species influence is represented by bar length on the histogram. (**b**) Dendrogram, with concentric circles denoting taxonomic levels (from phylum to genus). Each small circle on different classifications represents a taxonomic unit, scaled proportionally to its contribution (LDA score) in specific groups. Red and green dots denote core bacterial populations in respective models. (**c**) Heatmap correlating important bacterial genera in Oula sheep with functionally predicted KO pathways. (**d**) Heatmap correlating important bacterial genera in Hu sheep with functionally predicted KO pathways.

LEfSe analysis was performed on both sheep breeds to identify key taxonomic groups. Under the LDA > 3 criterion, 26 and 23 bacterial communities were identified as markers for Oula and Hu sheep, respectively (Figure 5a). All identified phyla predominantly belonged to Firmicutes, Bacteroidetes, Proteobacteria, Verrucomicrobiota, and Spirochaetota. Firmicutes and Bacteroidetes constituted the two dominant phyla. Significant differences in microbial community structures existed between Hu and Oula sheep. The Oula group exhibited significant enrichment in families associated with fiber degradation and energy metabolism, including Lachnospiraceae, Ruminococcaceae, and Butyricicoccaceae within Firmicutes. Notably, the family Akkermansiaceae (phylum Verrucomicrobiota)—known for mucin degradation and gut barrier regulation—and all taxa within Spirochaetota—implicated in fiber fermentation—were specifically enriched in the Oula group. Conversely, the Hu group exhibited significant enrichment of Christensenellaceae, Erysipelotrichaceae, and taxa within Proteobacteria, including the class Gammaproteobacteria and order Enterobacterales (Figure 5b). Correlation were detected between the microbial genera detected with differing abundances in LEfSe analysis and predicted KO pathways. including *Christensenellaceae_*R-7_group, *Akkermansia*, *Monoglobus*, *Succinivibrio*, *Coprococcus*, *Anaerostipes*, *Roseburia*, and *Anaerovibrio*, etc. These key microbial communities were subjected to Pearson correlation analysis with functionally differentiated KO pathways. The results indicate that in Hu sheep, the *Coprococcus*, which is positively correlated with acrosome integrity, is significantly positively correlated with the replication and repair (**) and translation (**) pathways. *Roseburia*—another genus positively correlated with acrosome integrity—showed a positive correlation with chemical structure transformation maps (*) and negative correlations with nervous system and folding, sorting, and degradation pathways (*). Anaerostipes was positively correlated with cell motility (*). Other correlation results are shown in Figure 5c. In Oula sheep, the *Akkermansia* genus, which is negatively correlated with the acrosome integrity rate, is significantly positively correlated with the chemical structure transformation maps (***), while it is negatively correlated with the pathways of cell motility (*), replication and repair, folding classification and degradation (*). *Family_*XIII-AD3011_group and UCG-009 are negatively correlated with carbohydrate metabolism (*) (Figure 5d).

### 3.6. Analysis of Potential Microorganisms Associated with Semen Parameters

**Figure 6:**
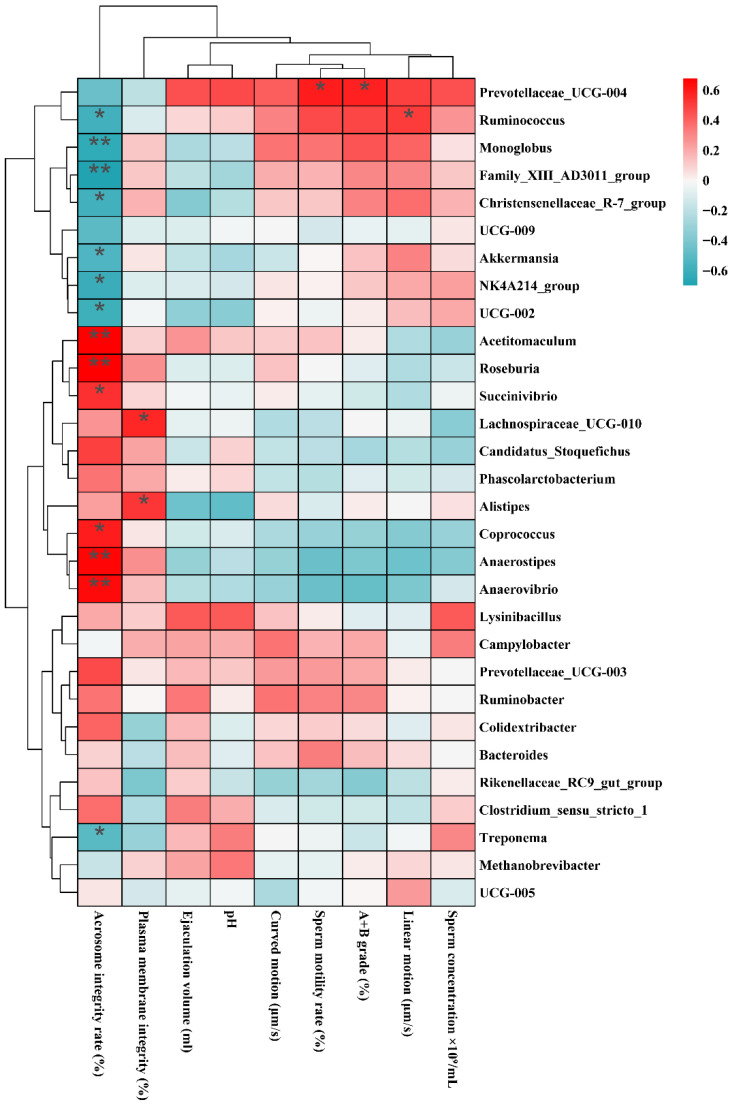
Correlation analysis between semen parameters and key microbial genera. Heatmap of correlations between two important sheep microbial genera and acrosome integrity rate, ejaculation volume, pH, curved motion, sperm motility rate, A + B grade (Progressive Motility, A is a fast-moving sperm, B is a slow-moving sperm, and A + B is a total forward moving sperm, %), linear motion, and sperm concentration. Significance levels are denoted by asterisks (*: *p* < 0.05, **: *p* < 0.01).

Correlation analysis between semen parameters and the top 30 important bacterial genera revealed (Figure 6) that *Acetitomaculum* (*r* = 0.68, *p* < 0.01), *Roseburia* (*r* = 0.67, *p* < 0.01), *Succinivibrio* (*r* = 0.53, *p* < 0.05), *Coprococcus* (*r* = 0.59, *p* < 0.05), *Anaerostipes* (*r* = 0.66, *p* < 0.01), and *Anaerovibrio* (*r* = 0.64, *p* < 0.01) showed significant positive correlations with acrosome integrity rate. *Ruminococcus* (*r* = −0.58, *p* < 0.05), *Monoglobus* (*r* = −0.64, *p* < 0.01), Family_XIII_AD3011_group, (*r* = –0.70, *p* < 0.01), *Christensenellaceae_*R-7_group (*r* = −0.59, *p* < 0.05), *Akkermansia* (*r* = −0.54, *p* < 0.05), *NK4A214_*group (*r* = −0.61, *p* < 0.05), UCC-002 (*r* = −0.60, *p* < 0.05), and *Treponema* (*r* = −0.52, *p* < 0.05) showed significant negative correlations with acrosome integrity rate. *Lachnospiraceae_UCG-010* (*r* = 0.56, *p* < 0.05) and *Alistipes* (*r* = 0.53, *p* < 0.05) were positively correlated with plasma membrane integrity rate. *Prevotellaceae_*UCG-004 (*r* = 0.59 and *r* = 0.57, *p* < 0.05) was positively correlated with sperm motility and A+B grade spermatozoa proportion. *Ruminococcus* (*r* = 0.51 and *r* = −0.58, *p* < 0.05) was positively correlated with sperm linear motility and negatively correlated with acrosome integrity rate.

## 4. Discussion

The gut microbiota, as an important mediator of host–environment interaction, plays a crucial role in the adaptation of animals to extreme habitats [12]. Multiple factors, including animal growth and developmental stage [13], genetic factors [14], dietary nutritional levels [15], and environmental conditions [7], can influence the gut microbiota. Previous studies have shown that the gut microbiota of indigenous animals on the plateau, such as Tibetan sheep and yaks, exhibits unique adaptive characteristics in terms of structural composition and metabolic function, which helps hosts efficiently obtain energy from low-quality diets [16,17]. However, there is currently a lack of in-depth research on whether this energy-first adaptation strategy has an impact on reproductive performance. Sperm acrosome integrity is an important indicator for evaluating semen quality and male reproductive capacity, and its formation and maintenance are regulated by various physiological factors. Based on this, this study compared the composition of fecal microbiota between indigenous Oula sheep and introduced Hu sheep, and analyzed its association with sperm acrosome integrity, aiming to explore the relationship between high-altitude adaptation and reproductive performance from the perspective of gut microbiota.

In this study, we observed distinct differences in fecal microbiota composition between Oula and Hu sheep, with notable enrichments in specific microbial taxa that were further associated with sperm acrosome integrity. Specifically, compared with introduced Hu sheep, the indigenous Oula sheep exhibited significantly higher alpha diversity indices (Simpson, Shannon, and Pielou_e), indicating greater diversity, richness, and evenness in their gut microbiota. As a native breed of the Qinghai–Tibet Plateau, the Oula sheep’s living environment is characterized by low oxygen, harsh conditions, and scarce high-quality forage. Previous studies have shown that animals such as Tibetan pigs, Tibetan chickens, Tibetan sheep, and yaks have evolved diverse and abundant gut microbial communities to enhance digestive efficiency and meet nutritional demands. For example, compared with domesticated yak, wild yak is exposed to more diverse dietary sources and complex ecological niches, resulting in their gut microbiota exhibiting higher species diversity, community complexity, and functional richness [7]. Compared with conventional broiler chickens, Tibetan chickens have evolved a more complex gut microbial community, alongside unique physiological adaptations that synergize with it [18]. Furthermore, Tibetan pigs exhibit richer and more diverse gut microbiota than pigs inhabiting low-altitude regions in southern Yunnan [19]. These disparities indicate that intense environmental selection pressures are key drivers of host-associated microbial community structure evolution. The present study demonstrated that, in comparison to the introduced Hu sheep, the indigenous Tibetan Oula sheep exhibited significantly higher alpha diversity in their fecal microbiota. Further analysis shows that Oula sheep were characterized by a significant enrichment of microbial taxa involved in fiber degradation (e.g., *Treponema*, Ruminococcaceae), mucin degradation (e.g., *Akkermansia*), and methanogenesis (e.g., *Methanobrevibacter*). Meanwhile, PCA and PERMANOVA analyses revealed significant differences in fecal microbial communities between Hu and Oula sheep, a finding validated by ANOSIM and MRPP methods. These findings indicate that Oula sheep not only have a higher diversity of gut microbiota, but their community composition also exhibits unique high-altitude adaptation characteristics. Correlation analysis further revealed that several of these enriched genera, including *Ruminococcus* and *Treponema*, were significantly negatively correlated with sperm acrosome integrity. There may be a potential association between gut microbiota and high-altitude adaptation and reproductive performance. These findings provide preliminary evidence for a potential association between the gut microbiota and the interplay between high-altitude adaptation and reproductive performance in ovine species. Differences in microbial community structure between breeds were also observed in pigs [20], Tibetan sheep [6], and goats [21].

Li et al. [16] investigated the rumen microbiota of native and introduced ruminants and found that during forage scarcity, rumen microbiota achieves efficient energy acquisition and nutrient utilization through enhanced fiber degradation capacity, increased volatile fatty acid (VFA) production, and reduced methane production. Firmicutes and Bacteroidetes promote fermentation and metabolism, degrade carbon sources, oligosaccharides, proteins, and amino acids, and enhance crude fiber digestion [22]. In this study, Oula and Hu sheep fecal microbiomes were dominated by Firmicutes and Bacteroidetes, this is consistent with previous views that these categories significantly affect the degradation and metabolic function of intestinal fibers in ruminants [23]. Moreover, Verrucomicrobiota was significantly higher in the Oula group than in the Hu sheep group. Previous studies have confirmed that in high-altitude regions, scarce forage resources lead to enrichment of the host’s Verrucomicrobiota. This phylum, particularly the genus *Akkermansia*, utilizes host intestinal mucins as an alternative carbon source, supplying energy to intestinal cells [24]. The relative abundance of the Firmicutes phylum in Oula sheep was significantly higher than in the Hu sheep group. This difference may reflect an adaptive response of gut microbiota to changes in the host’s nutritional environment. Bai et al. [25] conducted a metagenomic analysis of Tibetan antelope fecal microbiota and revealed abundant uncultured microbial groups, including *unidentified* Ruminococcaceae, *Christensenellaceae_*R-7_group, and *Prevotellaceae_*UCG-003, alongside *Bacteroides* and *Akkermansia*. These groups likely constitute core microbial functional clusters, degrading recalcitrant plant fiber substances. This study similarly observed the abundant presence of these uncultured microbial groups. Although the dominant bacterial genera remained consistent across both sheep breeds, we observed that the level of bacteria believed to have potential benefits for fiber degradation is significantly higher than that of Hu sheep. In particular, *Akkermansia*, *Methanobrevibacter*, and *unidentified* Ruminococcaceae were significantly more abundant than in Hu sheep. Previous studies have linked this group of bacteria to the breakdown of recalcitrant plant fibers in feed, suggesting that it may help Oula sheep digest poor quality diets and cope with harsh environments. However, further research is needed to confirm this functional level speculation. *Prevotella*, *Clostridium*, *Ruminobacter*, and *Treponema* all play critical roles in the degradation of crude fiber in two sheep breeds [26]. The present study revealed that the relative abundance of these fiber-degrading-associated microbiota was higher in Oula sheep (6.53%) than in Hu sheep (5.93%). Based on environmental factors and breed differences, we speculate that environmental conditions may be related to changes in the abundance of key fiber-degrading microorganisms, which may further affect the differences in crude fiber degradation and utilization efficiency between two sheep breeds.

The rumen microbial ecosystems of high-altitude yak and Tibetan sheep exhibit significant differences from their low-altitude relatives, domestic cattle and sheep. These differences manifest as markedly reduced methane production and higher VFA content [27]. This is considered as an adaptive evolutionary response to low oxygen and severe cold conditions in high-altitude environments [28]. Among these high-altitude native ruminants, bacterial groups such as Butyricicoccaceae, Anaerostipes, Coprococcus, and Ruminococcaceae exhibit high abundance. Butyricicoccaceae [29] can synthesize VFAs through metabolic processes, including organic nutrient chemical energy conversion and fermentation, producing formate, butyrate, and lactate. Genera such as *Anaerostipes* and *Coprococcus* [30] also participate in butyrate synthesis, supplying energy to intestinal epithelial cells. Spirochaetaceae, Ruminococcaceae, and *Ruminococcus* possess cellulolytic capabilities, degrading fibrous materials to generate VFAs such as butyrate and propionate. These VFAs help maintain an acidic intestinal environment, inhibit harmful bacterial growth, and support host energy metabolism [31]. While our study analyzed fecal microbiota, which primarily reflects hindgut communities, we observed consistent patterns such as enrichment of fiber-degrading taxa (Ruminococcaceae, *Treponema*) in Oula sheep. This congruence suggests that the adaptive signals may be detectable throughout the gastrointestinal tract, but it is necessary to use rumen sampling for confirmatory studies. Previous research [17] further revealed that multiple genes associated with VFA transport and absorption were upregulated in the rumen epithelial cells of native high-altitude Tibetan sheep. Moreover, their rumen microbiota exhibited richer VFA metabolic pathways compared with low-altitude introduced breeds, suggesting that plateau-adapted animals may possess more efficient VFA production capabilities. Similarly, the present study observed significantly higher abundance of Butyricicoccaceae in the gut of Oula sheep compared with that of Hu sheep. This is consistent with the inference that the former may have metabolic advantages in fiber fermentation and energy harvesting. Regarding cold stress adaptation research, Cheng et al. [32] compared gut microbial responses between Hulunbeier and Hu sheep during cold exposure. The author found that post-cold stress, the gut microbiota of Hulunbeier sheep exhibited increased abundance of *Treponema bryantii*, *Roseburia* sp. 499, and *Prevotella copri* and a marked enhancement in microbial functional pathways associated with propionic and butyric acid metabolism. The enrichment of these propionate- and butyrate-related microbial communities promote heightened propionic and butyric acid metabolism within the Hulunbeier sheep gut, thereby aiding energy conservation in cold environments. Along with the enhanced propionic acid/butyric acid metabolism exhibited by Hulunbuir sheep under cold stress, the dominance of fiber-fermenting bacteria such as the family Clostridiaceae in the gut microbiota of Oula sheep suggest that these microbial composition and functional differences may represent natural evolutionary adaptations to the high-altitude cold environment and specialized diet.

Methane production emissions result in energy loss from the diet. Increased VFAs can significantly suppress methane production by competing for hydrogen in the methane pathway [33], thereby inducing a “low-methane phenotype.” Xue et al. [34] identified microbial abundance differences between high- and low-feed-efficiency dairy cows through integrated metagenomic, transcriptomic, and metabolomic analyses. In low-efficiency cows, the *Methanobrevibacter* metabolic pathway exhibited heightened activity, suggesting a great loss of feed energy as methane. In the present study, high-altitude Oula sheep exhibited an elevated relative abundance of *Methanobrevibacter*, a finding consistent with the distinctive methanogenic microbial community structure observed in plateau ruminants. Among Tibetan sheep on the Qinghai–Tibetan Plateau, the dominant species within *Methanobrevibacter* is not *M. gottschalkii*, commonly found in low-altitude regions, but rather *M. millerae* [35]. This finding suggests that high-altitude adaptation may occur at the phylum, genus, and species levels of the microbiota through specific selection and enrichment. The dominant position of *M. millerae* in plateau sheep populations may result from host–microbe coevolution to adapt to high-fiber, low-nutrient pasture. Its specific metabolic functions and contribution to methane emissions warrant further investigation.

PICRUSt2 analysis predicted the difference in functional gene abundance between the two varieties. Compared with the Hu group, the Oula group showed a higher abundance of functional genes related to the “replication and repair”, “translation”, and “protein folding, classification, and degradation” pathways. These findings provide preliminary insights into the functional potential of fecal microbiota. The abundance of pathways related to protein and energy metabolism is consistent with previous research on Tibetan sheep, which may reflect the adaptation of microorganisms to low-protein, low-energy diets in high-altitude environments. Zhou et al. [36] reported that Tibetan sheep exhibit effective nitrogen utilization under these dietary conditions, which may be related to microbial metabolic capacity. Our observations are consistent with this pattern. In the future, further research combining metagenomics, transcriptomics, or metabolomics will be conducted to validate these functional predictions and elucidate their biological significance.

Efficient fiber degradation, VFA synthesis, and enrichment of protein/energy metabolic pathways are undoubtedly beneficial for the survival of Oula sheep in extreme environments. However, this study found that these energy acquisition-related microbial taxa (e.g., *Ruminococcus*, *Treponema*, *Akkermansia*) were significantly negatively correlated with sperm acrosome integrity. This phenomenon may reflect a trade-off between energy acquisition and reproductive investment during high-altitude adaptation. The enrichment of fiber-degrading bacteria represented by *Ruminococcus* and *Treponema* contributes to increased production of volatile fatty acids (VFAs) and optimized energy acquisition efficiency, serving as an adaptive strategy to cope with hypoxia and nutrient scarcity on the Qinghai–Tibet Plateau [17,35]. Previous studies have established that high-altitude hypoxia induces oxidative stress [37,38], which directly impairs sperm acrosome integrity by promoting lipid peroxidation of the sperm membrane [10]. Furthermore, the gut microbiota has been shown to influence host oxidative status through modulation of intestinal barrier function and inflammatory responses [39,40]. These lines of evidence provide a conceptual basis for linking gut microbiota, oxidative stress, and sperm quality in the context of high-altitude adaptation. There is reason to speculate that this microbial fermentation mode, while prioritizing energy supply, may entail trade-offs that affect sperm production through multiple pathways involving the gut–testis axis. One potential pathway involves sex hormones. Previous research [41] demonstrated that the abundance of the phylum Firmicutes in the gut is independently associated with host testosterone levels. In the present study, *Ruminococcus* (negatively correlated with acrosome integrity) and *Roseburia* and *Coprococcus* (positively correlated with acrosome integrity) all belong to the phylum Firmicutes. We speculate that the unique composition of Firmicutes in Oula sheep may differ in testosterone homeostasis compared to lowland breeds, which may affect androgen-dependent acrosome development. It is worth noting that a study in Rongchang boars also found that the genus Treponema was enriched in boars with low semen utilization, suggesting that the association between this genus and decreased sperm quality may be conservative across different species [42]. Comparative studies have revealed distinct antioxidant profiles between high-altitude and lowland sheep breeds. Wang [43] reported that Wugu sheep (high-altitude) exhibited significantly higher semen antioxidant enzyme activities (T-AOC, CAT, SOD) and lower MDA content than Hu sheep (low-altitude), suggesting enhanced antioxidant defense mechanisms in high-altitude-adapted sheep. Similarly, Li et al. [38] found that Tibetan sheep reared at 3800 m had significantly lower serum SOD activity than those at 2500 m, while FSH and testosterone levels showed no significant differences, indicating that oxidative stress pathways may be more sensitive to altitude than hormonal pathways. Interestingly, despite reduced SOD activity, sperm motility was maintained in high-altitude Tibetan sheep, suggesting the existence of compensatory adaptive mechanisms such as enhanced glycolysis and cAMP signaling. This is consistent with our observation that Oula sheep, despite having lower acrosome integrity, maintained other semen parameters comparable to Hu sheep. The higher abundance of *Akkermansia* in Oula sheep may be associated with degradation of the intestinal mucus layer, leading to increased intestinal permeability and facilitating translocation of bacterial products such as lipopolysaccharide (LPS) into the bloodstream. Previous studies have shown that lipopolysaccharide translocation is associated with systemic oxidative stress, making the acrosome structure of developing sperm more susceptible to oxidative damage—a mechanism discussed in the context of the gut–testis axis [40]. Wang et al. [10] demonstrated that reactive oxygen species (ROS)-induced oxidative stress directly impairs ram sperm acrosome integrity, and that antioxidant supplementation with chlorogenic acid significantly improved acrosome integrity while reducing ROS and MDA levels. The acrosome membrane is particularly vulnerable to oxidative damage due to its high content of polyunsaturated fatty acids, a mechanism also implicated in the gut–testis axis [40]. Additionally, a study in sheep revealed that gut microbiota dysbiosis can indirectly impair sperm function by influencing levels of neurotransmitters such as melatonin and dopamine, as well as glucocorticoids [44]. Based on this, we speculate that the enrichment of *Akkermansia* under high-altitude hypoxic conditions may exacerbate reproductive impairment through similar hormonal pathways, but its specific mechanism of action needs further research. The above findings are consistent with previous studies suggesting that the gut microbiota may remotely regulate host reproductive performance through the gut–testis axis. One of the pathways through which this axis acts involves the hypothalamic–pituitary–gonadal–axis: high-altitude hypoxic environments are associated with functional inhibition of the HPG axis, changes in sex hormone levels, and alterations in reproductive system function [37,45]. In this study, the sperm acrosome integrity of Hu sheep introduced from high-altitude areas was significantly higher than that of local Oula sheep, which may be related to the physiological effects of long-term exposure to extreme environments in Oula sheep. It is worth noting that the relative abundance of Euryarchaeota in the fecal microbiota of Oula sheep is significantly higher than that of Hu sheep, which may indicate that their intestinal microenvironment is in a relatively low oxygen state. Previous studies have shown that high-altitude hypoxia can alter the microbial composition of the body and promote the proliferation of anaerobic bacteria, and low oxygen and oxidative stress can affect the acrosome membrane of sperm, leading to a decrease in acrosome integrity [39,46]. Previous studies have confirmed that oxidative stress is closely related to semen quality. On this basis, this study proposes a possible regulatory mechanism from the perspective of gut microbiota: the gut microbiota may affect semen quality by regulating the host’s oxidative state. This mechanism may involve two interrelated paths: (i) balancing energy allocation-prioritizing survival needs while reducing energy investment in reproduction; and (ii) microbial-mediated intestinal barrier dysfunction leading to increased systemic oxidative stress and potentially further amplified by high-altitude hypoxic environments. It should be emphasized that although this hypothesis is consistent with our correlation data and the existing literature, it still needs further verification.

This study has several limitations. These include a relatively limited sample size (n = 8 per group), which may limit our ability to detect differences in certain semen parameters, the use of fecal microbiota as a proxy that may not fully represent the entire gastrointestinal tract, and the absence of key intermediate physiological measurements. Future studies with larger sample sizes, integrated multi-omics approaches, and comprehensive physiological assessments are warranted to validate and extend our findings.

## 5. Conclusions

In this study, we characterized the fecal microbial composition of Oula and Hu sheep raised in the Qinghai–Tibet Plateau region. Significant differences in fecal microbial diversity, richness, and taxonomic composition were observed between the two breeds. Oula sheep exhibited higher alpha diversity and evenness, as well as enrichment of bacterial taxa associated with key physiological functions. These included fiber-fermenting genera such as *Ruminococcus* and *Treponema*, the mucin-degrading bacterium *Akkermansia*, and butyrate-producing Butyricicoccaceae. Correlation analysis revealed that several of these enriched taxa were negatively associated with sperm acrosome integrity. These findings indicate that host genetics and long-term environmental exposure shape distinct fecal microbial profiles in sheep, and suggest a potential link between high-altitude-adapted microbiota and reproductive traits. Further studies integrating metagenomics, metabolomics, and physiological measurements are needed to explore the underlying mechanisms connecting gut microbiota and sperm quality in high-altitude livestock.

## Figures and Tables

**Table 1:** Statistics of basic sequence information generated by amplicon sequencing.

Sample	RawPE Reads	Combined Tags	Qualified Tags	Effective Tags	Effective%
OL1	83,110	82,905	82,623	78,542	94.50
OL2	71,310	71,153	70,972	67,504	94.66
OL3	65,750	65,639	65,496	62,902	95.67
OL4	54,088	53,975	53,764	51,765	95.71
OL5	73,197	73,033	72,770	70,689	96.57
OL6	105,769	105,532	105,205	101,300	95.77
OL7	67,179	67,011	66,748	63,714	94.84
OL8	104,218	103,965	103,548	98,155	94.18
HU1	115,336	115,073	114,669	97,067	84.16
HU2	99,976	99,752	99,389	95,391	95.41
HU3	113,904	113,637	113,198	107,973	94.79
HU4	71,549	71,397	71,163	67,428	94.24
HU5	82,618	82,457	82,207	78,524	95.04
HU6	105,998	105,748	105,368	100,065	94.40
HU7	106,673	106,444	106,089	100,497	94.21
HU8	98,984	98,784	98,409	93,115	94.07

**Table 2:** Analysis of differences in community structure among breeds.

Method	OL-HU	
ANOSIM	R-value	0.46974
	*p*-value	<0.001
MRPP	A	0.06174
	*p*-value	<0.001

## Data Availability

The original contributions presented in this study are included in the article. Further inquiries can be directed to the corresponding author.
